# Multitarget Effects of Nrf2 Signalling in the Brain: Common and Specific Functions in Different Cell Types

**DOI:** 10.3390/antiox13121502

**Published:** 2024-12-10

**Authors:** Elisa Navarro, Noemí Esteras

**Affiliations:** 1 Neurochemistry Research Institute, Department of Biochemistry and Molecular Biology, School of Medicine, Complutense University of Madrid, 28040 Madrid, Spain; 2 CIBERNED, Network Center for Biomedical Research in Neurodegenerative Diseases, 28040 Madrid, Spain; 3 Instituto Ramón y Cajal de Investigación Sanitaria (IRYCIS), 28034 Madrid, Spain; 4 Group of Neurodegenerative Diseases, Hospital Universitario 12 de Octubre Research Institute (imas12), 28041 Madrid, Spain; 5 Department of Clinical and Movement Neurosciences, UCL Queen Square Institute of Neurology, London WC1N 3BG, UK

**Keywords:** Nrf2, brain, neurons, astrocytes, microglia, antioxidants, mitochondria, inflammation, neurodegeneration

## Abstract

Nuclear factor erythroid 2-related factor 2 (Nrf2) is a crucial regulator of cellular defence mechanisms, essential for maintaining the brain’s health. Nrf2 supports mitochondrial function and protects against oxidative damage, which is vital for meeting the brain’s substantial energy and antioxidant demands. Furthermore, Nrf2 modulates glial inflammatory responses, playing a pivotal role in preventing neuroinflammation. This review explores these multifaceted functions of Nrf2 within the central nervous system, focusing on its activity across various brain cell types, including neurons, astrocytes, microglia, and oligodendrocytes. Due to the brain’s vulnerability to oxidative stress and metabolic challenges, Nrf2 is emerging as a key therapeutic target to enhance resilience against oxidative stress, inflammation, mitochondrial dysfunction, and demyelination, which are central to many neurodegenerative diseases.

## 1. Nrf2-Keap1 Signalling Pathway

The nuclear factor erythroid 2-related factor 2 (Nrf2) is a transcription factor encoded by the *NFE2L2* gene, known to be a master regulator of multiple cytoprotective pathways by inducing the expression of a variety of genes, including antioxidant, detoxifying, and intermediary metabolic enzymes [1].

**Figure 1:**
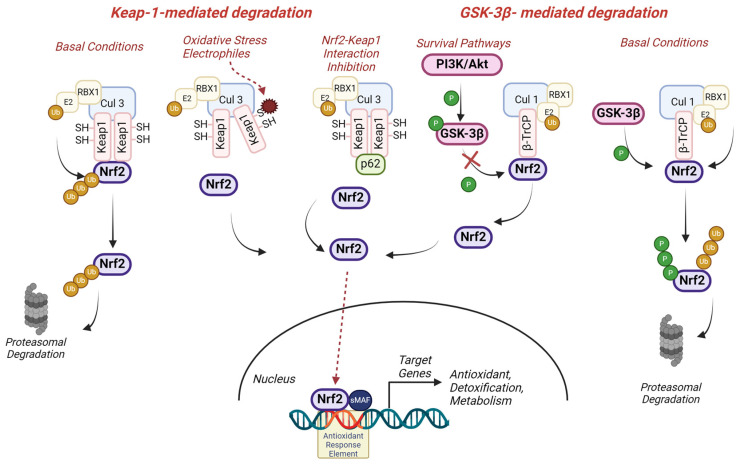
Molecular regulation of Nrf2. The transcription factor Nrf2 is a master regulator of multiple cytoprotective pathways by inducing the expression of antioxidant, detoxification, and intermediary metabolic enzymes. Under basal conditions, Nrf2 is targeted for continuous proteasomal degradation by different ubiquitin-ligase systems. The cullin 3 (Cul3) RING-box 1 (RBX1) ligase complex mediated degradation employs the Kelch-like ECH-associated protein 1, Keap1, as a substrate adaptor. Keap1 is a cysteine-rich adaptor protein that binds Nrf2, facilitating its ubiquitination and targeting it for degradation under basal conditions. Keap1 acts as an oxidative stress sensor, and in the presence of oxidative signals or electrophiles, Keap1’s highly reactive Cys residues become oxidised, leading to a conformational change that prevents Nrf2 binding, allowing its stabilisation and translocation to the nucleus. Nrf2 stabilisation can also be achieved by several molecules, such as p62 or pharmacological inhibitors, which prevent Nrf2 binding to Keap1. The β-TrCP/Cul 1 ubiquitin ligase complex mediates the degradation of Nrf2 in a mechanism involving the glycogen synthase kinase 3 (GSK3β). In basal conditions, GSK3β-mediated phosphorylation of Nrf2 targets the protein for degradation through this system. When GSK3β is inhibited, such as after its phosphorylation by the PI3K/Akt survival pathway, Nrf2 degradation does not occur, allowing its stabilisation and translocation to the nucleus. By either way, once in the nucleus, Nrf2 binds to the antioxidant response elements (ARE) present in the promoter of its target genes, inducing their transcriptional activation. Created in Biorender.

Nrf2 levels are tightly regulated, and under normal physiological conditions, Nrf2 is maintained at low levels in the cytoplasm through its interaction with Kelch-like ECH-associated protein 1 (Keap1) (Figure 1). Keap1 is a cysteine-rich protein that acts as a substrate adaptor for the Cullin (Cul3)-dependent E3 ubiquitin ligase complex, binding and targeting Nrf2 for ubiquitination and subsequent proteasomal degradation [2]. This constitutive degradation pathway ensures the tight regulation of Nrf2 activity, preventing excessive activation in the absence of stress stimuli. Keap1 acts as a crucial sensor for oxidative stress and, upon exposure to oxidative signals or electrophiles, the oxidation of Keap1’s highly reactive cysteine residues leads to conformational changes in the protein that disrupt its interaction with Nrf2, allowing Nrf2 to escape from Keap1-mediated degradation, accumulating and translocating to the nucleus [3]. Nrf2 stabilisation can also be achieved by other mechanisms that facilitate Keap1 degradation or impair the Nrf2-Keap1 binding, such as the interaction of the autophagy-related protein p62 with the Nrf2-binding domain of Keap1 [4]. Additionally, other ubiquitin-ligase systems have also been described to control Nrf2 degradation independently of Keap1, like the β-TrCP/Cul 1 system (Figure 1). The glycogen synthase kinase-3 beta (GSK3-β) acts as a modulator of this route by phosphorylating Nrf2 and targeting it for degradation [5]. Since GSK3-β activity, and therefore Nrf2 degradation, is regulated by survival pathways such as PI3K/Akt or Wnt, the interplay between Nrf2 and GSK3-β represents a key regulatory axis in receptor-mediated signal transduction, influencing cell survival under conditions of oxidative damage and increased metabolic demands.

Either way, once in the nucleus, Nrf2 heterodimerizes with small Maf proteins and binds to antioxidant response elements (ARE) located in the promoter regions of its target genes (Figure 1). This binding triggers the transcriptional activation of a diverse array of genes encoding antioxidant enzymes (e.g., superoxide dismutase, catalase, glutathione peroxidase), phase II detoxification enzymes (e.g., NAD(P)H oxidoreductase 1 (NQO1), glutathione S-transferases, heme-oxygenase I (HO-1)), and other cytoprotective proteins [6,7].

### Pharmacological Activation of Nrf2-Keap1 Signalling Pathway

Given its protective role, Nrf2 activation is an attractive therapeutic strategy for diseases characterised by mitochondrial dysfunction and oxidative stress [8,9]. Various pharmacological agents can induce Nrf2, as reviewed in [10]. Most of these activators are electrophilic compounds that covalently modify Keap1’s cysteine residues, causing structural changes that prevent Nrf2 degradation. Examples include sulforaphane, TBE-31, dimethyl fumarate (DMF), omaveloxolone, and several natural compounds. Efforts are underway to develop non-electrophilic compounds that specifically inhibit the Nrf2-Keap1 interaction without covalent modification [10]. Some Nrf2 activators are in different stages of clinical development, and a few are already approved as disease-modifying treatments for various conditions [10]. Notably, fumaric acid ester derivatives, such as DMF, are approved for treating relapsing-remitting multiple sclerosis with long-term safety and efficacy [11,12], and omaveloxolone has been recently authorised in the USA and Europe for the treatment of Friedreich’s ataxia [13]. The fact that both are neurological disorders, highlights the key cytoprotective role of Nrf2 in the brain, both in physiology and also as a pharmaceutical target.

## 2. Nrf2, the Cytoprotective Defence in the Brain

**Figure 2:**
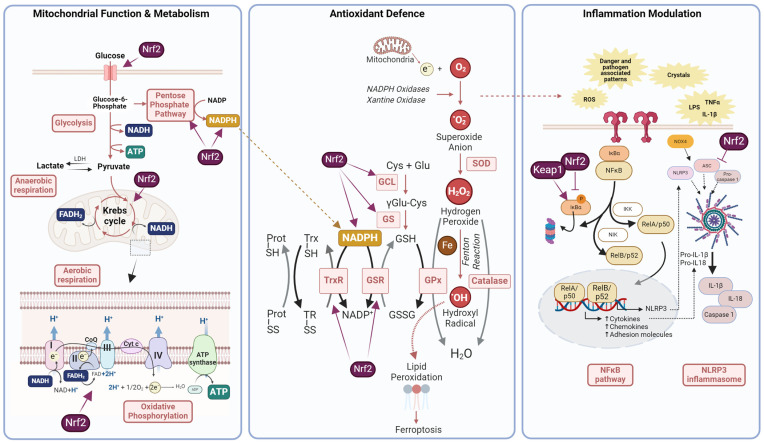
Main cytoprotective pathways of Nrf2 in the brain. Nrf2 orchestrates several essential cellular functions in the brain, including mitochondrial function and metabolism, antioxidant defence, and the modulation of the inflammatory response. **Left panel:** The brain primarily relies on glucose as an energy source. Glucose is taken up by cells via glucose transporters and metabolised through glycolysis, generating pyruvate, ATP, and NADH. Alternatively, glucose can be processed via the pentose phosphate pathway, which is critical to produce the cofactor NADPH. Pyruvate can either be converted into lactate during anaerobic respiration or enter the mitochondria to fuel the Krebs cycle. The Krebs cycle, a series of enzymatic reactions, produces NADH and FADH2, which donate electrons to the mitochondrial electron transport chain for ATP generation through oxidative phosphorylation. Nrf2 modulates glucose metabolism by increasing glucose uptake, enhancing the activity of enzymes involved in its metabolism and improving the efficiency of the Krebs cycle, thereby increasing the availability of the substrates NADH and FADH_2_ for mitochondrial respiration and, subsequently, ATP production. **Middle panel:** Enzymatic sources, such as xanthine oxidase and NADPH oxidase, along with non-enzymatic sources like electron leakage from the mitochondria, trigger the production of reactive oxygen species (ROS). This occurs primarily through the conversion of O_2_ into the superoxide anion (O_2_^•−^) which is rapidly converted into hydrogen peroxide (H_2_O_2_), either spontaneously or by the action of superoxide dismutase (SOD). In the presence of iron, H_2_O_2_ can generate the hydroxyl radical (•OH) through the Fenton reaction. The hydroxyl radical (•OH) is extremely reactive and can initiate lipid peroxidation in cellular membranes, ultimately triggering ferroptosis. To limit the damage caused by ROS, H_2_O_2_ is detoxified into water (H_2_O) by various antioxidant pathways, such as catalase and glutathione peroxidases (GPx), which catalyse the reduction of H_2_O_2_ (and other peroxides) using glutathione (GSH) as a co-substrate. GSH is synthesised in the cytosol from its constituent amino acids by the sequential actions of glutamate-cysteine ligase (GCL) and glutathione synthetase (GS). Another crucial player in antioxidant defence is the thioredoxin (Trx) system, which reduces disulfide bonds in oxidised proteins (Prot-SS). Cellular redox reactions are ultimately driven by NADPH, which is used to reduce oxidised thioredoxin (Trx-SS) and oxidised glutathione (GSSG) through thioredoxin reductase (TrxR) and glutathione reductase (GSR), respectively, regenerating reduced thioredoxin (Trx-SH) and GSH. Among other mechanisms, Nrf2 modulates the cellular antioxidant defence by upregulating the expression of key enzymes involved in the synthesis and regeneration of GSH (GCL, GS, GR) and the thioredoxin (TrxR) systems, while also enhancing the production of NADPH through the activation of the pentose phosphate pathway. **Right panel:** Various pro-inflammatory signals, including lipopolysaccharide (LPS), cytokines, damage-associated patterns, and reactive oxygen species (ROS), can activate specific receptors on microglial cells, thereby initiating the NF-κB and inflammasome pathways. Within the NF-κB pathway, IKK (in the canonical pathway) or NIK (in the non-canonical pathway) phosphorylate IKBα, leading to its degradation by the proteasome. This degradation results in the release of RelA/p50 or RelB/p52, which then translocate to the nucleus to activate the transcription of inflammatory mediators such as cytokines, chemokines, and adhesion molecules. Moreover, it also permits the expression of NLRP3 and pro-IL-1β, coordinating the priming step in the NLRP3 (NOD-like receptor protein 3) inflammasome pathway. Afterwards, in the activation step, the NLRP3 protein, the adaptor protein ASC, and pro-caspase-1 assemble the inflammasome complex and activate caspase-1, IL-1β, and IL-18. The crosstalk between Nrf2 and NF-κB pathways underscores the intricate balance between antioxidant defences and inflammatory responses in maintaining brain homeostasis. Created in Biorender.

The brain is an exceptionally energy-demanding organ due to its unique functions, such as sustaining neuronal activity and synaptic transmission. These energy requirements make the brain a highly active metabolic organ which represents only about 2% of body weight but consumes up to 20% of the total oxygen in the body, as it relies mostly on glucose oxidation for ATP production. As a consequence, a high amount of reactive oxygen species (ROS) are produced as a byproduct of mitochondrial respiration, which need to be neutralised to avoid oxidative damage (Figure 2). This is essential, since the brain is particularly rich in polyunsaturated lipid membranes, prone to oxidation, and free iron, which acts as a catalyst in the formation of ROS, initiating a cycle of oxidative damage and metabolic failure finally leading to neuronal death [14] (Figure 2).

In this context, endogenous Nrf2 plays a pivotal role as one of the key cytoprotective mechanisms in the brain, acting as a master regulator of brain energy metabolism and defence against oxidative stress and inflammation, among other things (Figure 2). Nrf2 is differentially expressed in different brain regions and cell types within the brain, being more abundant in glial cells (including oligodendrocytes, astrocytes and microglia) rather than in neurons [15] (https://www.proteinatlas.org/ENSG00000116044-NFE2L2/single+cell+type, accessed on 13 September 2024). In the following sections, we will explore current knowledge on several aspects of Nrf2 function across the different brain cell types within the central nervous system (CNS), its impact on brain health, and its potential implication in neurodegenerative diseases.

### 2.1. Nrf2 in Neurons

Neurons are highly specialised cells responsible for electro-chemical signalling transmission and are considered to consume about 80% of the brain’s energy. These exceptionally high energy requirements are largely due to the activity of Na^+^/K^+^ ATPase pumps, which are essential for restoring the ion gradients after excitatory synaptic signalling and action potentials propagation [16]. To meet these needs, neurons rely heavily on oxidative metabolism, making mitochondria a key component of their energy production. This intense metabolic activity also results in a higher generation of ROS due to the leakage of electrons out of the mitochondrial electron transport chain in an oxygen-rich environment (Figure 2). When not properly neutralised, ROS accumulation might lead to oxidative stress to which neurons are particularly vulnerable for different reasons, such as the high susceptibility of neuronal lipid membranes to oxidation [14]. Therefore, the balance between large energy production and the management of oxidative stress is critical for maintaining neuronal health and preventing neurodegenerative damage.

#### 2.1.1. Nrf2, Neuronal ROS and Neuronal Development

In this scenario, it appears paradoxical that neurons have a relatively weak antioxidant defence compared to other cell types in the brain. Isolated neurons are known to exhibit low levels of catalase and glutathione, two key endogenous antioxidants, both regulated by Nrf2, which also shows minimal basal activity in these cells [17]. In fact, although Nrf2 is considered ubiquitous, results from multiple single cell RNA-seq and single nucleus RNA-seq datasheets consistently show that the *NFE2L2* gene is repressed in most of the mature neurons in the CNS [18]. This downregulation appears to occur at the epigenetic level, where the hypo-acetylation of the histones associated with *NFE2L2* promoter prevents gene transcription by limiting chromatin accessibility [18,19]. Not only Nrf2 mRNA is downregulated in mature neurons, but also Nrf2 protein, which appears to be more unstable when compared to other brain cells, partially due to a parallel increase in Keap1 and Cul1 expression [20]. In agreement, the mRNA and protein levels of some of Nrf2 target genes such as NQO1 or HO-1, among others, are also lower in neurons than in other cell types, suggesting an overall weaker Nrf2 system in this cell type [18,20].

*NFE2L2* repression occurs during the development and differentiation of neuronal precursors, when Nrf2 mRNA levels decline and Keap1 levels increase, shutting down the activity of this transcription factor during development [18]. In contrast, Nrf2 is highly expressed in embryonic stem cells and plays a key role in their self-renewal capacity, proliferation, and differentiation [21]. Likewise, neuronal stem cells and neuronal progenitor cells also show elevated Nrf2 mRNA levels [19]. Indeed, Nrf2 plays an important role in their function both during the early stages of development and in the regulation of the activity of the neuronal stem cells in the adult brain [22]. These cells are responsible for the generation of new neurons and astrocytes during adult neurogenesis in specific brain niches such as the subventricular zone. It is known that their activity and neurogenic potential declines with age, and this occurs in correlation with an age-dependent decline in Nrf2 activity [23]. Indeed, modulation of Nrf2 appears to restore their neurogenic and proliferative potential [24]. However, even when Nrf2 activity is crucial for the function of precursor cells, its inactivation becomes critical for proper neuronal maturation as differentiation progresses, since redox signalling is essential for neuronal development [19]. Although a disbalance in their levels is strongly associated with the onset of many diseases, ROS also play an important role in physiology and cell signalling. Among the different processes they regulate, an optimal level of brain ROS is necessary for the proper maturation of neurons [25]. Among others, redox signalling regulates different pathways essential for development, such as Wnt, which can be potentiated by ROS [26]. Nrf2, through its potent antioxidant capacity, prevents redox-dependent Wnt activation, suggesting that its shut-off during development is essential to allow for a boost of Wnt signalling in this period [19]. Indeed, forcing Nrf2 expression during early differentiation impairs neuronal maturation, including neurite outgrowth and synaptogenesis [19]. It is still not clear why Nrf2 levels remain low in mature neurons, despite their higher antioxidant demands, but it is known that ROS signalling controls many aspects of neuronal function [27,28], so it could be possible that a basal chronic activation of Nrf2 in neurons could interfere with these processes. However, epigenetic de-repression of neuronal Nrf2 in mature neurons appears to enhance its cytoprotective effects under damaging circumstances [29].

#### 2.1.2. Nrf2, Neuronal ROS and Neurodegenerative Disorders

It has also been argued that reduced Nrf2 expression in neurons in physiological conditions could be due to an insufficient rate of ROS to trigger Nrf2 activation. Nevertheless, neuronal expression of Nrf2 also appears to be low in some neurodegenerative conditions associated with increased oxidative stress such as Alzheimer’s disease (AD) [30] or multiple sclerosis [31], according to single nucleus RNA sequencing profiles. Conversely, studies at the subcellular level in human brain tissues from patients with various neurodegenerative conditions reveal distinct patterns depending on the disease. In AD, Nrf2 predominantly exhibits a cytoplasmic localization in hippocampal neurons affected by the pathology [32], whereas in Parkinson’s disease (PD), neurons in the substantia nigra show a pronounced nuclear localization of Nrf2, likely indicating a compensatory response to elevated oxidative stress [32,33]. This has also been found in in vitro models of other neurodegenerative conditions. For example, induced neurons derived from amyotrophic lateral sclerosis (ALS) patients with the C9orf72 mutation showed higher Nrf2 nuclear location and activity, indicating an activation of the Nrf2 pathway under oxidative stress conditions [34]. This partial activation of Nrf2 appeared insufficient to mitigate the elevated levels of ROS present in these cells. However, pharmacological activators of Nrf2, such as DMF, were able to significantly reduce oxidative stress [34]. This finding suggests that, although endogenous Nrf2 activation was insufficient to protect neurons, external activation through compounds like DMF effectively enhanced the antioxidant response, offering improved defence against oxidative damage. This neuroprotective effect has been further demonstrated in several animal models of different disorders, either by genetic or pharmacological activation of the Nrf2 pathway: for detailed reviews, see [7,35].

#### 2.1.3. Nrf2 and Neuronal Mitochondria

As previously mentioned, the processes involved in synaptic transmission significantly account for the majority of ATP consumed by neurons. Mitochondria are the primary source of neuronal ATP and play a critical role in maintaining synaptic homeostasis, by acting as localised calcium buffers during pre- and post-synaptic events, which involve large influxes of cytosolic calcium [36]. Mitochondrial calcium homeostasis is closely linked to energy production; increased calcium uptake boosts ATP synthesis, helping neurons adapt to their energy needs. Additionally, calcium modulates the movement and positioning of mitochondria within neurons, directing them to areas with higher energy demands such as the axon terminals. Therefore, proper mitochondrial function is crucial for optimal neuron activity, and it is well established that Nrf2 regulates various aspects of mitochondrial activity, both in normal physiology and as a potential therapeutic target in pathological conditions [37].

Although low compared to other cell types, basal neuronal Nrf2 expression has been described as playing an important role in the neuronal mitochondrial function, as described in in vitro and animal models of genetical Nrf2 deficiency. Isolated primary neurons of Nrf2 knockout mice present increased oxidative stress and mitochondrial bioenergetics dysfunction, including impaired respiration and a less efficient activity of the Krebs cycle, leading to reduced levels of mitochondrial NADH and ATP, compared to wild-type neurons [38,39]. This is accompanied by defects in neuronal dendritic arborization and synaptic gene expression and is associated with cognitive deficits in the Nrf2-deficient aged mice [39,40]. In contrast, in isolated neurons of Keap1 knockdown mice, genetic activation of Nrf2 enhances respiration efficiency by increasing the availability of substrates for the electron transport chain (ETC), leading to higher ATP production in the mitochondria compared with wild-type neurons [38] (Figure 2). Indeed, primary cultures from this mice model show that genetic Nrf2 activation enhances glucose uptake into neurons, which is preferentially metabolised in the glycolytic/oxidative phosphorylation pathway to produce energy [41].

Beyond its physiological function, the stimulation of neuronal glucose metabolism by Nrf2 underlies some of the neuroprotective effects that its pharmacological activation has shown in different pathological contexts. Neurons are very vulnerable to mitochondrial dysfunction and energy deprivation, which are a common hallmark of different neurodegenerative disorders. Among them, PD is characterised by an impairment of mitochondrial respiration due to insufficient substrate availability for the ETC, especially at complex I. This leads to reduced ATP production, which is particularly detrimental to the dopaminergic neurons in the *substantia nigra*, a region severely impacted by the disease. These mitochondrial effects, which resemble those observed in Nrf2 deficiency, can be reverted by the pharmacological activation of Nrf2, which has been shown to enhance mitochondrial substrate provision for the ETC [42]. Activation of Nrf2 with sulforaphane or omaveloxolone (RTA-408) was shown to reverse bioenergetic deficits in cultured neurons within a PINK1-deficient PD model and prevent neuronal death, confirming the role of Nrf2 activation in supporting mitochondrial bioenergetics in neurons [43]. The mitochondrial and bioenergetic effects of omaveloxolone have also been observed in Friedreich’s ataxia, a hereditary, slowly progressive ataxia characterised at the molecular level by impaired mitochondrial function due to reduced substrate availability for ETC Complex I [44]. Pharmacological activation of Nrf2 with omaveloxolone increased the availability of NADH for mitochondrial complex I in cerebellar granular neurons of mouse models of the disease and restored the mitochondrial respiration, preventing neuronal death [44]. The neuroprotective effects of omaveloxolone were also demonstrated in both in vivo and in vitro models, preventing the onset of seizures [45]. Prolonged seizure-like activity induces synchronised calcium oscillations, which lead to mitochondrial depolarization, depletion of ATP and GSH, and neuronal damage. Administration of omaveloxolone after the onset of status epilepticus increased the reduced form of glutathione, GSH (γ-L-glutamyl-L-cysteinylglycine), and ATP levels, preventing associated neuronal damage and the spontaneous seizures that typically develop in the weeks following status epilepticus [45]. Crucially, Nrf2 activation provided neuroprotection by enhancing mitochondrial bioenergetic function and supplying neuronal ATP, helping to meet the increased energy demands caused by high-frequency calcium oscillations during seizures. Overall, these findings suggest that modulating mitochondrial bioenergetics through Nrf2 activation is a promising therapeutic strategy for neurodegenerative diseases, given the significant energy demands of neurons and their dependence on mitochondria [9].

In addition to improving neuronal mitochondrial function and preventing oxidative stress, numerous in vivo studies have highlighted various aspects of the neuroprotective effects of the pharmacological activation of Nrf2 across different neuropathological conditions [46,47]. However, it is also important to consider the role of non-neuronal cells in this process. Indeed, it has been argued that the Nrf2-mediated neuronal protection can be attributed to astrocytic Nrf2 activation [48], which appears to play a key role in the process, as will be discussed in the next section.

### 2.2. Nrf2 in Astrocytes

Astrocytes, along with neurons, originate from neuroepithelial radial glia, through a process governed by different signals that determine the neural or glial fate of their progenitors. They are the most abundant glial cells in the human adult brain and play a crucial role in maintaining the CNS homeostasis [49]. Among other functions, such as regulating neurotransmitter recycling or synapse maturation, they are known to play a role in providing support for neuronal functions, including energy homeostasis and antioxidant defence. In addition, in response to brain injury, astrocytes undergo molecular and functional modifications which can have both beneficial and harmful effects on neuronal function and survival [49].

In contrast to neurons, astrocytes are known to produce elevated levels of antioxidants, with a major role for the glutathione, thioredoxin, and catalase systems [50] (Figure 2). Likewise, the Nrf2 pathway appears to be highly active in astrocytes, both at the mRNA and protein levels and by the abundance of some of its target genes [20]. In this case, astrocytic Nrf2 activity is maintained throughout the maturation process, suggesting that the molecular mechanisms implicated in their differentiation are different to those of neurons, and probably less dependent on ROS signalling [17].

Neuronal and astrocytic metabolic profiles also differ significantly as astrocytes are highly glycolytic and rely less on oxidative phosphorylation. Moreover, astrocytes are thought to supply neurons with lactate as an energy substrate via the astrocyte-neuron lactate shuttle [51]. Similarly to neurons, Nrf2 activation increases glucose uptake in astrocytes and plays a role in modulating its metabolism, as shown in primary cultures from the Keap1 knockdown mice model [41]. Once internalised, glucose is metabolised by two main metabolic pathways: either glycolysis, oriented to energy production, or the pentose phosphate pathway, which among others generates NADPH, a cofactor needed by many antioxidant enzymes. When glucose availability is limited, Nrf2 has been shown to prioritise the metabolism of glucose for energy production in astrocytes, with a smaller contribution to support redox reactions [41]. This highlights the importance of the role of astrocytic Nrf2 in supporting brain energy metabolism, both in physiology and under conditions of energy deprivation.

#### 2.2.1. Nrf2 and Neuronal–Astrocytic Crosstalk

Neuronal–astrocytic communication is key to couple neuronal needs and astrocytic support, and Nrf2 has been said to mediate some of the signalling cascades that allow this crosstalk. Among them, the molecular events occurring during synaptic transmission are inevitably linked to an increase in neuronal ROS, and coupling of neuronal activity with Nrf2 activation in astrocytes supports neurons with an adequate antioxidant defence [52]. During neurotransmission, glutamate released has been shown to act not only in the post-synaptic neurons but also to stimulate metabotropic glutamatergic receptors in astrocytes [53]. Activation of astrocytic glutamatergic receptors leads to the translocation of Nrf2 to the nucleus [20,53] and allows the transcription of genes involved in glutathione metabolism in astrocytes [54]. These GSH precursors can be then released by astrocytes and used by the neighbouring neurons to synthetize GSH, thus protecting them from oxidative stress [55] and coupling neuronal activity to astrocytic-induced neuroprotection [53]. Nevertheless, neuronal activity has also been linked to the activation of some neuroprotective Nrf2 target genes independently of astrocytes and the Nrf2 pathway, which suggests the cooperation of different pathways in the process [56,57].

Adaptive astrocytic neuroprotective responses have also been shown to be triggered by other stimuli. For example, Bell et al. showed that exposure of co-cultures of neurons and astrocytes to mild levels of oxidative stress (sub-toxic hydrogen peroxide levels) led to a neuroprotective response that depended on the activation of Nrf2 pathway in astrocytes [58]. However, other studies have shown that endogenous astrocytic hydrogen peroxide does not activate the Nrf2 pathway, yet it can confer neuroprotection through Nrf2-independent mechanisms [59]. Mitochondrial dysfunction has also been found to trigger Nrf2 activation in astrocytes [60], while astrocytic-specific Nrf2 overexpression is able to protect neurons against mitochondrial-induced neurotoxicity in an in vivo model of mitochondrial complex II inhibition [60].

Indeed, experiments in in vivo models of different neurodegenerative conditions such as PD [48,61], ALS [62], spinal cord injury [63], or vascular cognitive impairment [64] support the role of astrocytic Nrf2 in neuroprotection. These studies show how selective overexpression of Nrf2 in astrocytes under a glial fibrillary acidic protein (GFAP) promoter protected dopaminergic neurons, motor-neurons, spinal cord, and white matter, respectively, from degeneration [48,61,62,63,64].

In addition to endogenous Nrf2, experiments in cellular cultures have shown that Nrf2 inducers preferentially target astrocytes [65]. Therefore, when considering the neuroprotective role of Nrf2 pharmacological activation in the brain, it is important to pay attention to the specific role of astrocytes in the different neurodegenerative disorders: for a detailed review, see [66].

#### 2.2.2. Nrf2 and Astrocytic Function: Its Role in Inflammation

Some of the molecular pathways involved in the physiological and therapeutic astrocytic-mediated neuroprotection have to do with the role of astrocytes as mediators of the brain’s inflammatory response, with Nrf2 playing a key role in modulating this process [67,68]. Upon activation, Nrf2 reduces the expression of pro-inflammatory cytokines, thus shifting astrocytes toward an anti-inflammatory phenotype. This action limits excessive inflammation, which could otherwise lead to astrocytic dysfunction and contribute to pathological conditions such as neuroinflammation, which is a hallmark of various neurodegenerative diseases. Indeed, it is known that specific subpopulations of reactive astrocytes induce neurotoxicity in different neurological disorders [69], and single cell RNAseq-based studies identified a reduced Nrf2 activity within those astrocytic populations as the underlying cause of the neuroinflammation observed in a multiple sclerosis model [70]. Therefore, astrocytic Nrf2 is a critical component in protecting against neurodegenerative disorders, where chronic neuroinflammation plays a central role in disease progression [71].

Nevertheless, the modulation of the inflammatory response in the CNS is not exclusive to astrocytes but is also a key function of other glial cells such as the microglia, and the interplay between them. The molecular pathways linked to this aspect of Nrf2 function will be described in detail in the following sections.

### 2.3. Nrf2 in Microglia

Microglia, the brain’s resident immune cells, play crucial roles in both health and disease. They originate from yolk sac progenitors, migrate to the developing brain, and become isolated by the blood–brain barrier (BBB). Microglia have a complex “sensome” composed of surface receptors that allow them to detect and react to a variety of situations [72]. In the attempt to react and respond to physiological and pathological contexts, microglia acquire and coexist in various dynamic states named as phenotypes [73,74]. As resident efficient macrophages of the brain, microglia undergo crucial functions including, although not limited to, neurogenesis, synaptic remodelling, the release of soluble factors, tissue repair, or phagocytosis [74]. Microglia display a higher expression of Nrf2 compared to neurons, as they show higher ARE activity and more Nrf2 transcripts [75].

The role of Nrf2 in modulating the innate immune system was first addressed using models of acute inflammation. One of the first pieces of evidence arises with a model of experimental sepsis where the absence of Nrf2 is associated with more severe damage, higher lethality, and increased pro-inflammatory markers [76]. Using the lipopolysaccharide (LPS) model of acute inflammation, several authors have also shown how the absence of Nrf2 results in higher inflammatory response (both in vitro and in vivo), shown by an increased cytokine release and changes in F4/80 immunostaining and morphology within the brain, pointing to a response mediated by microglia [77]. Moreover, in the MPTP model of PD, the absence of Nrf2 leads to a higher cytokine release and a reduction in the expression of anti-inflammatory markers. This situation was associated with a higher neuronal vulnerability in basal ganglia [78]. Similarly, in a model of tauopathy, the microgliosis elicited by mutant tau can be prevented pharmacologically by Nrf2 inducers such as DMF [79], whereas the effect is lost in Nrf2-/- animals. Several mechanisms reviewed below contribute to the effect of Nrf2 in controlling inflammation.

#### 2.3.1. Nrf2 Inhibits NF-κB Signalling Pathway

The molecular mechanism underlying this anti-inflammatory effect implies different players, including the transcription factor nuclear factor κB (NF-κB) multicomplex (Figure 2). NF-κB is a pivotal mediator in the innate and adaptive immune response, orchestrating the expression of a number of pro-inflammatory cytokines and chemokines. Classical pro-inflammatory stimuli, such as LPS, TNF, or IL-1β, activate the canonical pathway. This pathway leads to the activation of the IKK complex, which promotes the phosphorylation of IκBα and degradation via the proteasome pathway. This allows the release of the RelA/p50 dimer, which translocates to the nucleus and participates in the expression of pro-inflammatory genes, including cytokines, chemokines, and adhesion molecules, among others (Figure 2). In the non-canonical pathway, the NIK (NF-κB inducible kinase) phosphorylates the IκKα complex, leading to the subsequent release of the p52/RelB complex. The effect of Nrf2 on the NF-κB signalling pathway was established by the observation that Nrf2-deficient animals showed increased NF-κB activation (as well as downstream mediators such as cytokines and adhesion molecules) in a model of traumatic brain injury [80]. Indeed, Nrf2 deficiency promotes IκBα phosphorylation and degradation, thus permitting a higher expression of NF-κB and therefore promoting inflammation [81]. Keap1 has been shown to be implicated in this process, as it is also responsible for the ubiquitination and subsequent degradation of IKKβ. Therefore, in the absence of Keap1 a stabilisation of IKKβ occurs which also leads to maintained inflammation [82]. It should be noted that this regulation is not unidirectional. In addition, NF-κB can also inhibit or prevent Nrf2 induction by several mechanisms including the competition with the coactivator CBP or recruiting HDAC3, which promotes histone hypoacetylation and impedes Nrf2 signalling [83]. Indeed, a number of anti-inflammatory agents can activate Nrf2 by inhibiting the NF-κB pathway [84]. Besides its action on NF-κB, studies using chromatin immunoprecipitation (ChIP)-seq have revealed that Nrf2 binds near genes induced during inflammation (IL-6, IL-1β) and prevents their expression [85].

#### 2.3.2. Nrf2 Prevents the NLRP3 Inflammasome Formation

Another mechanism by which Nrf2 controls the inflammatory response is by interfering in the inflammasome formation. Inflammasomes serve as sensors of inflammatory signals, such as infectious agents or cell damage, and in response to these signals they trigger an innate immune response [86]. One of such inflammasome is the NOD-like receptor protein 3 (NLRP3) inflammasome, a multiprotein cytoplasmic complex which is crucial in the regulation of the inflammatory response [87]. Furthermore, inflammasome formation can induce cell death through pyroptosis, which also contributes to the propagation of inflammation and damage. Three components form this multicomplex structure: the NLRP3 protein which acts as sensor, an adaptor protein named ASC, and an effector pro-caspase-1 protein [88] (Figure 2). Its activation requires two subsequent steps: first, a priming step responsible for the transcription of NLRP3, pro-IL-1β and pro-IL-18, which is mediated by the translocation of NFκB to the nucleus. Subsequently is the activation step, in which occurs the assembly of the inflammasome complex that finally activates caspase-1. This leads to the maturation and secretion of pro-inflammatory cytokines, including IL-1β and IL-18. Although classical triggers of NLRP3 formation are danger and pathogen molecular patterns, intracellular ROS can also induce its activation. As both, NLRP3 and Nrf2 can respond to oxidative stress, the accumulation of ROS acts as the link in the interplay between both signalling pathways. Under a situation of oxidative stress, the thioredoxin-interacting protein binds to NLRP3 and triggers its assembly [89]. Another protein implicated in the inflammasome activation under oxidative conditions seems to be the NADPH oxidase (NOX) 4 (NOX4), which is an important source of ROS, catalysing the production of superoxide anion and hydrogen peroxide (Figure 2). The blockade of this enzyme has been demonstrated to prevent NLRP3 formation in oxidative conditions, as NOX4-deficient animals and glial cells fail to produce IL-1β or IL-18 in different models associated with inflammatory and oxidative damage [90]. Thus, both NLRP3 and Nrf2 are activated under the accumulation of oxidative stress and Nrf2 would elicit its antioxidant effect, counteracting NLRP3 formation. Indeed, it has been widely reported how Nrf2 inducers can block NLRP3 assembly in microglia, including sulforaphane [91], DMF [92], or melatonin [93,94], among others. Similarly, but with an opposite approach, Nrf2-deficient macrophages have been shown to upregulate NLRP3 and the subsequent production of cleaved caspase-1 [95]. The inhibitory role of Nrf2 on NLRP3 inflammasome formation seems to happen in the priming step and can also derive from the role that Nrf2 has in blocking NFκB signalling as well as from its antioxidant capacity. As reviewed in a previous section, Nrf2 has a role in inhibiting NFκB signalling, consequently blocking the expression of genes needed for NLRP3 formation. Nrf2 target genes such as HO-1 and NQO-1 have been demonstrated to be implicated in this process. For example, NQO1 overexpression in macrophages through the activation of Nrf2 derives in a reduced expression of NLRP3 and caspase-1, and consequently, reduced release of IL-1β [95].

Although all previous research points to an inhibitory role of Nrf2 on NLRP3 formation, other authors have also shown that Nrf2 is needed for an appropriate activation. In an in vivo model of atherosclerosis, the activation of Nrf2 by cholesterol crystals leads to NLRP3 formation, whereas the ablation of Nrf2 in this model would be preventing the inflammatory damage [96]. Similarly, it has been reported how Nrf2-deficient macrophages show a defect in the maturation and secretion of IL-1β and caspase-1 [97], pointing to Nrf2 as a positive regulator in the formation of this complex.

#### 2.3.3. Nrf2 Regulates Phagocytosis

Besides the role of Nrf2 in the control of inflammation, this transcription factor is also crucial for another function associated with phagocytes, which is the clearance of apoptotic cells, mediated by a process named as efferocytosis. The role of Nrf2 controlling this process has been shown in a myriad of contexts, both in the CNS and the periphery.

Experiments performed in the context of multiple sclerosis, where the uptake of myelin debris is fundamental, led to the observation that Nrf2 was also implicated in the process of phagocytosis. Using peripheral macrophages and primary microglia, Grajchen and collaborators showed that Nrf2 is necessary for the uptake of myelin debris [98]. In the cited paper, they show that Nrf2 controls the expression of the scavenger receptor CD36. In the absence of Nrf2, there is a deficit in the expression of this receptor, which affects phagocytosis, specifically the subprocess of uptake of debris. Moreover, Nrf2 deficient microglia also showed impaired phagocytosis in basal conditions or after stimulation with alpha-synuclein as a model of PD [99] and tauopathy [100]. The molecular mechanism behind this dysfunction may imply mediators such as Axl or Mer, which showed a decreased expression in Nrf2-deficient microglia. Analogously, the use of Nrf2 inducers such as sulforaphane increases CD36 expression, which promotes phagocytosis of red blood cells by brain microglia in a model of intracerebral haemorrhage [101].

Nevertheless, efferocytosis is a complex mechanism that not only implies the uptake, but also the digestion and elimination of the cargo [102] and the involvement of Nrf2 at these levels still needs to be elucidated.

#### 2.3.4. Nrf2 as a Regulator of Immunometabolism in Microglia

As reviewed before, microglia counteract a myriad of functions, such as phagocytosis, proliferation, or cytokine release, which require continuous metabolic adjustments to support their activities. Whereas homeostatic microglia rely on oxidative phosphorylation for ATP production, they undergo metabolic reprogramming during inflammatory conditions. This shift favours anaerobic pathways, which, although less efficient, provide a more rapid energy supply to meet the high demands of microglial activation and produce intermediates necessary for cytokine production [103]. A key player in all this immunometabolism regulation is itaconate, a mitochondrial metabolite generated by microglia and macrophages in response to inflammatory stimuli to control damage [104]. The production of itaconate is catalysed by immune-responsive gene 1 (IRG1) in the mitochondrial matrix [105]. Pro-inflammatory stimuli such as LPS or interferons activate the expression of IRG1 and itaconate, which inhibits succinate dehydrogenase (or complex II), limiting ROS production and promoting an anti-inflammatory phenotype in myeloid cells [104]. Interestingly, Nrf2 also plays a role in this process, as itaconate has been shown to react with cysteine residues of Keap1, activating Nrf2 and promoting the expression of downstream genes [106]. Thus, IRG1 and itaconate have an anti-inflammatory and protective role in microglia, which is lost in the absence of Nrf2 [107].

Besides its role regulating metabolic reprogramming, Nrf2 is essential for maintaining mitochondrial functioning. Danger-associated molecular patterns produce a microglial response that commences inflammation and ROS production. As phagocytic cells, microglia express high levels of NOX, which produces superoxide radicals. Thus, microglial ROS are primarily produced by this enzyme and are important for shaping microglial phenotypes and host defence. Different cell surface receptors in the microglia such as toll-like receptors (TLRs), complement receptor 3 (CR3), purinergic receptors, or CD36 lead to the NOX-mediated oxidant production [108]. Among the different NOX subtypes, NOX2 seems to be highly expressed in microglia, localised in the plasma membrane, mostly associated with lipid rafts [108], followed by NOX4. The production of ROS by NOX is necessary for microglial functions such as phagocytosis or inflammation, and they act as secondary messengers that may propagate inflammatory states and damage associated phenotypes [109]. Nevertheless, it is important that antioxidant pathways such as Nrf2 modulate this oxidative environment to prevent deleterious propagation (Figure 2). Thus, Nrf2 is essential to counteracting oxidative damage, as reviewed in the previous sections, regulating mitochondrial antioxidant defence in microglia to prevent ROS accumulation, and enhancing NADPH supply, as reviewed elsewhere [110].

#### 2.3.5. Nrf2 Prevents Microglia from Ferroptosis

Ferroptosis is a mechanism of programmed cell death associated with a large accumulation of iron and lipid peroxidation, firstly described by Dixon and collaborators [111]. Microglia adjust iron transport and metabolism based on inflammatory signals, accumulating iron during inflammation by sequestering intracellular iron released from heme catabolism and extracellular iron taken up through transporters like the divalent metal transporter (DMT1) [112]. In this context, there is an increase in oxidative stress and lipid peroxidation which ultimately leads to this mechanism of programmed cell death. Ferroptosis process in microglia contributes to the release of pro-inflammatory signals and affects neighbouring cells; thus, the inhibition of ferroptosis in microglia may serve as a target for neuroprotection. In this context, many downstream genes of Nrf2 are directly linked to the regulation of ferroptosis including GPX4 (GSH peroxidase 4) or SLC7A11 (12-channel transmembrane protein transporter vector family 7 member 11) [113], this former promoting the generation of GSH. Moreover, as Nrf2 promotes an antioxidant environment, it reduces the sensitivity to ferroptosis. This mechanism has been shown in microglial cells, where the activation of Nrf2 promotes ferroptosis resistance, attenuating the inflammatory response [114].

Nevertheless, the downstream effector of Nrf2, HO-1, may play a deleterious role in the context of ferroptosis. Pro-inflammatory stimuli such as LPS, as well as ageing or pathology increases the expression of HO-1, which contributes to iron overload and ferroptosis, as it will be described in the following section. Indeed, it has been shown how the ablation/inhibition of HO-1 in microglia as well as the use of iron chelators can be protective in models associated with neuroinflammation [115,116].

#### 2.3.6. Role of Microglia Heme-Oxygenase I in the Function of Nrf2

In the control of microglial functions elicited by Nrf2, the role of HO-1 is noteworthy [117]. HO-1 is an inducible 32 kDa protein that catalyses the degradation of heme group, releasing free iron (Fe^2+^), carbon monoxide (CO), and biliverdin (BV) (rapidly converted to bilirubin). Among the transcriptional network regulated by Nrf2, the expression of the *Hmox1* gene is under Nrf2 regulation, among other factors. HO-1 is ubiquitously expressed and localises within the cells, especially in the endoplasmic reticulum and the cytosol [118], although it can also be associated with other intracellular membranes including in plasma caveoles and mitochondria [119]. HO-1 has canonical effects (directly derived from the byproducts generated by its enzymatic activity) and non-canonical effects which are not correlated with its enzymatic activity. In both cases, HO-1 has been shown to counteract inflammation. Whereas heme group has pro-inflammatory functions, the byproducts of its degradation via HO-1 (BV, CO and Fe^2+^) have shown direct anti-inflammatory effects [120,121]. Indeed, free Fe^2+^ and bilirubin (which derive from BV) inhibit NF-κB signalling and the production of pro-inflammatory cytokines. The non-canonical effects include protein–protein interaction (as HO-1 can directly interact with HO-2) and subcellular localization (i.e., translocation to the nucleus and other organelles), where its enzymatic activity is affected [122].

Multiple pieces of evidence point to HO-1 as a key factor regarding the beneficial roles of Nrf2 in microglia. Indeed, the blockade of this enzyme prevents the anti-inflammatory and antioxidant effects elicited by Nrf2 inductors either in microglial or organotypic cultures [123,124] and its absence prevents mitochondrial biogenesis in glial cells [125]. These beneficial effects have clinical consequences, as the induction of microglial HO-1 has been reported to provide protection in a model of brain haemorrhage [125], whereas the protection exhibited by Nrf2 induction in a photothrombotic model of stroke is lost in animals lacking HO-1 [126]. Nevertheless, an overactivation of this enzyme can also derive in deleterious effects, mainly due to Fe^2+^ overload, as described in the previous section [115]. Indeed, in ageing and AD samples (both human and mice) there is an increased expression of HO-1 predominantly in microglia [127], which could be a compensatory mechanism although it can also trigger harmful effects. Among them, it has been described how long-term overactivation of HO-1 can induce tau aggregation [128,129], increase microgliosis [130], and result in cognitive decline in models of tauopathy and AD [131]. These beneficial and deleterious effects derived from HO-1 have been reviewed elsewhere [132]. Altogether, these results highlight the importance of a tight regulation of Nrf2 and its downstream targets, as a sustained overexpression can lead to deleterious effects.

### 2.4. Nrf2 in Oligodendrocytes

Finally, although far less explored, Nrf2 also regulates the functionality of oligodendrocytes, which are defined as the myelin-producing cells of the CNS. They are crucial for myelination and myelin regeneration in the CNS and are essential for reducing energy consumption and enhancing neuronal signal transmission. Oligodendrocytes also support neurons metabolically by transporting necessary metabolites and play a role in modulating neuroinflammation through interactions with microglia. They are characterised by high metabolic rates, and consume large amounts of oxygen and ATP, which ultimately leads to the production of ROS [133]. Moreover, in the myelination process, many of the enzymes are iron-dependent, thus having large stores of iron, which can ultimately produce lipid peroxidation [133] (Figure 2). Taken all together, oligodendrocytes are especially vulnerable to metabolic energy supply and oxidative stress, highlighting the potential importance of the Nrf2 system in this specific cell type. Indeed, among the different brain cell types they express the highest levels of Nrf2 according to transcriptomic analyses [15], Human Cell Atlas). Here, we will describe the current knowledge of Nrf2 in oligodendrocyte functionality.

#### 2.4.1. Metabolic Support and Antioxidant Defence of Nrf2 in Oligodendrocytes

As stated before, oligodendrocytes are metabolically active cells, which makes them especially vulnerable to oxidative damage. In this context, it has been shown, in an oligodendroglial cell line subjected to sodium azide as mitochondrial inhibitor, how Nrf2 activation through the knockdown of Keap1 partly prevented mitochondrial depolarization and increased metabolic activity. Opposite results were obtained when Nrf2 was knocked down [134]. Likewise, the use of pharmacological activators of Nrf2 in an oligodendroglial cell line protected from cell death elicited by hydrogen peroxide, inducing the synthesis of GSH and counteracting oxidative stress [135]. The oxidative damage associated with mitochondrial blockade in oligodendrocytes is connected with the expression of endoplasmic reticulum stress response, which has been demonstrated to also be mediated by Nrf2 [136]. Not only does Nrf2 reduce the oxidative damage through the activation of antioxidant defences, but it also promotes mitochondrial biogenesis through activation of PGC-1α [137], permitting a higher metabolic rate which is essential for oligodendrocyte differentiation.

#### 2.4.2. Nrf2 Participates in Myelination

Some studies have addressed the potential role of Nrf2 in the demyelination/remyelination process, mostly in the context of multiple sclerosis. Most of the studies presented in this section rely on the cuprizone model, which produces a transitory de-myelinization associated with oligodendrocyte loss. Using this model, it has been shown that the absence of Nrf2 produces an increased vulnerability [138]. Similarly, different Nrf2 inducers have shown protection against demyelinating insults [139,140]. Nevertheless, the mechanism involved is still elusive and might not be related (at least exclusively) to the role of Nrf2 in oligodendrocytes but to other cell types such as microglia and astrocytes. In this vein, it has been recently shown that during remyelination, astrocytes downregulate Nrf2 pathway, turning their metabolism towards cholesterol biosynthesis [141], which they export to oligodendrocytes to regulate their survival and remyelination. Indeed, these authors use a model that constitutively overexpresses Nrf2 in astrocytes (GFAP-Nrf2) and they show an impairment in remyelination [141]. On the contrary, opposite results have been obtained in other studies, where the activation of Nrf2 in the astrocytes using GFAP-specific Keap1 deletion animals showed lower oligodendrocyte loss and demyelination [142]. These different approaches highlight the importance of further exploring this pathway and demonstrate the importance of a tight control on Nfr2 activity to maintain brain homeostasis and prevent damage.

Myelin is composed mainly of lipids; thus, lipid homeostasis is key in the biology of oligodendrocytes. In this context, metabolomic studies showed that treatment of oligodendrocytes with DMF produced a reduction in most of the lipids (such as phosphatidylcholine, sphingomyelin, or free fatty acids) at the time-points that it exerted antioxidant and neuroprotective effects [143]. Authors speculate that the reduction in lipid synthesis could be an initial response, where all antioxidant enzymes need to be synthesised, whereas chronic treatment would switch towards metabolic reprogramming, implying higher lipid synthesis for myelination.

Besides the molecular pathway, the fact that DMF is the first approved Nrf2-inducer in clinic and is used for the treatment of multiple sclerosis demonstrates the importance of this factor for promoting myelination.

## 3. Conclusions and Future Perspectives

The transcription factor Nrf2 plays a pivotal role in maintaining brain health by regulating mitochondrial function, antioxidant defence, inflammation, and myelination, among other functions. Its activation across different brain cell types—neurons, astrocytes, microglia, and oligodendrocytes—demonstrates a complex yet interdependent role in cellular homeostasis and neuroprotection. Neurons, which are the cells most vulnerable to energy deprivation and oxidative stress, surprisingly present the lowest Nrf2 levels within the different cellular subtypes. However, even despite its low levels, Nrf2 still plays a vital role in supporting the mitochondrial function, mitigating oxidative stress, and preserving energy production in mature neurons. In astrocytes, it enhances antioxidant defence mechanisms, such as glutathione synthesis, which protects not only the astrocytes themselves but also adjacent neurons. Both astrocytes and microglia rely on Nrf2 for balancing their pro-inflammatory and anti-inflammatory responses, preventing chronic neuroinflammation while promoting tissue repair. Oligodendrocytes also benefit from Nrf2 activation, where it plays a role in the differentiation process and myelination, contributing to efficient neuronal signal transmission.

Despite these findings, there remain significant gaps in understanding the cell type-specific regulation of Nrf2 and its therapeutic potential in neurodegenerative diseases such as Alzheimer’s and Parkinson’s disease, as well as in demyelinating disorders like multiple sclerosis. Future research should focus on elucidating the distinct Nrf2-dependent pathways within each cell type under both physiological and pathological conditions. Furthermore, the development of targeted Nrf2 modulators could offer new therapeutic strategies tailored to specific cell types or disease states. However, more detailed studies are required to unravel its multifaceted roles in different brain cell types and its broader impact on neurodegenerative and demyelinating diseases.

## Abbreviations

AD—Alzheimer’s disease, ALS—amyotrophic lateral sclerosis, ARE—antioxidant response elements, BBB—blood–brain barrier, BV—biliverdin, CNS—central nervous system, CO—carbon monoxide, CR3—complement receptor 3, Cul—cullin, Cys—cysteine, DMT1—divalent metal transporter, DMF—dimethyl fumarate, ETC—electron transport chain, Fe^2+^—free iron, GFAP—glial fibrillary acidic protein, GSH—reduced glutathione (γ-L-glutamyl-L-cysteinylglycine), GPX4—GSH peroxidase 4, GSK3-β—glycogen synthase kinase-3 beta, HO-1—heme-oxygenase 1, IRG1—immune-responsive gene 1, Keap1—Kelch-like ECH-associated protein 1, LPS—lipopolysaccharide, NF-κB—nuclear factor κB, NIK—NF-κ B inducible kinase, NLRP3—NOD-like receptor protein 3, NOX—NADPH oxidase, NQO1—NAD(P)H oxidoreductase 1, Nrf2—nuclear factor erythroid 2-related factor 2, PD—Parkinson’s disease, ROS—reactive oxygen species, SLC7A11—12-channel transmembrane protein transporter vector family 7 member 11, TLRs—toll-like receptors.
